# Endothelial Glycocalyx Degradation Patterns in Sepsis-Associated Pediatric Acute Respiratory Distress Syndrome: A Single Center Retrospective Observational Study

**DOI:** 10.1177/08850666231200162

**Published:** 2023-09-06

**Authors:** Colin J. Sallee, Joseph A. Hippensteel, Kristen R. Miller, Kaori Oshima, Andrew T. Pham, Robert P. Richter, John Belperio, Yamila L. Sierra, Andreas Schwingshackl, Peter M. Mourani, Eric P. Schmidt, Anil Sapru, Aline B. Maddux

**Affiliations:** 1 Department of Pediatrics, Division of Pediatric Critical Care Medicine, David Geffen School of Medicine at University of California Los Angeles and Mattel Children's Hospital, Los Angeles, CA, USA; 2 Department of Medicine, Division of Pulmonary Sciences and Critical Care Medicine, University of Colorado Anschutz Medical Campus, Aurora, CO, USA; 3 Department of Pediatrics, Section of Pediatric Critical Care, University of Colorado School of Medicine and Children's Hospital Colorado, Aurora, CO, USA; 4 Department of Medicine, Division of Pulmonary and Critical Care Medicine, Harvard Medical School and 2348 Massachusetts General Hospital, Boston, MA, USA; 5 Department of Pediatrics, Division of Pediatric Critical Care Medicine, 9967 University of Alabama at Birmingham Heersink School of Medicine, Birmingham, AL, USA; 6 Department of Medicine, Division of Pulmonary Critical Care and Sleep Medicine, David Geffen School of Medicine at University of California Los Angeles and Ronald Reagan Medical Center, Los Angeles, CA, USA; 7 Department of Pediatrics, Division of Pediatric Critical Care Medicine, University of Arkansas for Medical Sciences and Arkansas Children's Hospital, Little Rock, AR, USA

**Keywords:** glycocalyx, proteoglycan, glycosaminoglycan, acute respiratory distress syndrome, mechanical ventilation, sepsis, pediatric intensive care, pediatrics

## Abstract

**Background:**

Sepsis-associated destruction of the pulmonary microvascular endothelial glycocalyx (EGCX) creates a vulnerable endothelial surface, contributing to the development of acute respiratory distress syndrome (ARDS). Constituents of the EGCX shed into circulation, glycosaminoglycans and proteoglycans, may serve as biomarkers of endothelial dysfunction. We sought to define the patterns of plasma EGCX degradation products in children with sepsis-associated pediatric ARDS (PARDS), and test their association with clinical outcomes.

**Methods:**

We retrospectively analyzed a prospective cohort (2018-2020) of children (≥1 month to <18 years of age) receiving invasive mechanical ventilation for acute respiratory failure for ≥72 h. Children with and without sepsis-associated PARDS were selected from the parent cohort and compared. Blood was collected at time of enrollment. Plasma glycosaminoglycan disaccharide class (heparan sulfate, chondroitin sulfate, and hyaluronan) and sulfation subtypes (heparan sulfate and chondroitin sulfate) were quantified using liquid chromatography tandem mass spectrometry. Plasma proteoglycans (syndecan-1) were measured through an immunoassay.

**Results:**

Among the 39 mechanically ventilated children (29 with and 10 without sepsis-associated PARDS), sepsis-associated PARDS patients demonstrated higher levels of heparan sulfate (median 639 ng/mL [interquartile range, IQR 421-902] vs 311 [IQR 228-461]) and syndecan-1 (median 146 ng/mL [IQR 32-315] vs 8 [IQR 8-50]), both *p *= 0.01. Heparan sulfate subtype analysis demonstrated greater proportions of *N*-sulfated disaccharide levels among children with sepsis-associated PARDS (*p = *0.01). Increasing *N-*sulfated disaccharide levels by quartile were associated with severe PARDS (n = 9/29) with the highest quartile including >60% of the severe PARDS patients (test for trend, *p *= 0.04). Higher total heparan sulfate and *N-*sulfated disaccharide levels were independently associated with fewer 28-day ventilator-free days in children with sepsis-associated PARDS (all *p < *0.05).

**Conclusions:**

Children with sepsis-associated PARDS exhibited higher plasma levels of heparan sulfate disaccharides and syndecan-1, suggesting that EGCX degradation biomarkers may provide insights into endothelial dysfunction and PARDS pathobiology.

## Introduction

Acute respiratory distress syndrome (ARDS) is a complex lung disease resulting from the intersection of several aberrant biological pathways including epithelial injury, microvascular endothelial dysfunction, and dysregulated inflammation.^1,2^ Mortality remains between 20% and 40% in adults and children, and despite promising preclinical data, there are no effective targeted therapies that improve outcomes.^3,4^ Current clinical definitions of ARDS in adults and children identify a highly heterogeneous group of patients that differ by underlying pathobiology and responses to therapies.^5-8^ Efforts to subgroup patients by shared pathobiological mechanisms may identify targets for precision-based therapeutic strategies.

Increasingly, the endothelial glycocalyx (EGCX) has been implicated in the pathogenesis of a number of acute and chronic illnesses (eg, sepsis, trauma, coronavirus disease-19, diabetes, and atherosclerosis).^9,10^ The EGCX is a ubiquitous gel-like apical extracellular matrix that lines the luminal surface of all vascular endothelial cells. It is comprised of endothelial membrane-bound proteoglycans (PGs) covalently linked to glycosaminoglycans (GAGs).^11,12^ The layer is crucial to maintaining organ and vascular homeostasis, and accordingly, demonstrates different morphologies concordant with the functions of the organs and capillaries in which it is found.^13^ The pulmonary EGCX is distinct from the systemic vascular beds due to its vast surface area, thickness exceeding 1.67 µm (compared to 0.67 µm in systemic vessels), and central role in maintaining pulmonary endothelial barrier function.^14,15^ The EGCX acts to selectively restrict fluid and protein permeability, enhancing capillary oncotic pressure and influencing fluid filtration as well as opposing leukocyte-endothelial interactions.^16,17^ Accordingly, disruption of the pulmonary EGCX by sepsis mediators (eg, cytokines) can manifest clinically as ARDS, where the pulmonary endothelium transforms into an active pro-inflammatory phenotype resulting in vascular hyperpermeability, edema formation, and leukocyte infiltration.^18-20^ Moreover, circulating EGCX degradation products are capable of influencing inflammatory signaling processes distant from the initial site of injury, underscoring the significance of endothelium-directed insults in sepsis-induced ARDS pathobiology.^21,22^

Given that disruption of the EGCX may be causally related to the microvascular endothelial dysfunction characteristic of sepsis-associated ARDS, human studies investigating the EGCX in sepsis-associated adult and pediatric ARDS (PARDS) are needed.^23^ Indeed, PGs and GAGs shed into circulation may serve as biomarkers of EGCX degradation that inform ARDS pathobiology. While evidence for the clinical relevance of these biomarkers are accumulating in adult ARDS, there is a paucity of data regarding the role of EGCX degradation in sepsis-associated PARDS. In this exploratory analysis, we sought to characterize the plasma patterns of EGCX degradation in critically ill children mechanically ventilated with sepsis-associated PARDS. We hypothesized that children with sepsis-associated PARDS would demonstrate increased fragmentation of endothelial-derived plasma GAGs and PGs, and that higher plasma levels of GAG and PG fragments would be associated with adverse clinical outcomes.

## Materials and Methods

### Design/Setting/Patients

We conducted a retrospective analysis of a prospectively enrolled cohort of children receiving invasive mechanical ventilation at a single academic pediatric intensive care unit (PICU) at Children's Hospital Colorado (CHCO) from 2018 to 2020 (Colorado IRB #18-0436). The parent cohort included children ≥1 month and <18 years of age at enrollment receiving ≥72 h of invasive mechanical ventilation who were expected to survive to discharge. Exclusion criteria included: tracheostomy in place at time of PICU admission, limitations to support, admission to CHCO or another inpatient facility for 7 days prior to intubation, or intubation at another facility >48 h prior to transfer to CHCO.

Eligible patients’ parent/guardians were approached within 7 days of initiation of invasive mechanical ventilation to obtain written informed consent. Consented patients were offered the option to have blood collected and were the focus of this analysis. Blood was collected on day of consent. Among patients with available plasma, a convenience sample of children with and without PARDS was identified from the parent cohort. PARDS diagnosis was based on the Pediatric Acute Lung Injury Consensus Conference-2 (PALICC-2) criteria (acute insult, chest X-ray with infiltrate(s), pulmonary edema not otherwise explained by fluid overload or cardiovascular dysfunction, and hypoxemia).^24^ Subsequently, children with sepsis-associated PARDS were classified by two person-adjudication (CJS and ABM) from the cohort diagnosed with PARDS. Using established criteria, sepsis was defined as having a known or suspected infection from a pulmonary or nonpulmonary source as the primary etiology of respiratory failure.^6,25,26^ This group was compared to children mechanically ventilated for neurologic failure or a nonpulmonary procedure/surgery from the cohort without PARDS. These patients served as a critically ill comparator group whose disease processes were not suggestive of lung injury and/or sepsis. The de-identified analyses were deemed IRB exempt (UCLA IRB #22-001375). All procedures followed ethical guidelines determined by an institutional responsible committee on human experimentation and the Helsinki Declaration.

### Data Collection

We collected demographics (age, sex, and weight), comorbidities (presence of chronic pulmonary conditions, neurologic disease, immunocompromise [including oncologic disease or hematopoietic cell transplantation]), elements specific to PARDS (risk factors, ventilator data, chest imaging, and oxygen saturations), illness severity (pediatric risk of mortality score [PRISM-III]), and organ dysfunction scores (pediatric logistic organ dysfunction score [PELOD-2]).^27,28^ Data collection focused on the first week of invasive mechanical ventilation with ventilator data and organ dysfunction scores recorded once daily at 0800 up to 7 days unless the patient died or was discharged from the PICU (whichever occurred first). Oxygenation saturation index (OSI: mean airway pressure * Fio_2_/Spo_2_ * 100) defined the severity of hypoxemia and risk stratified PARDS categories into severe versus mild/moderate. Children with OSI ≥5 were diagnosed with PARDS; children with OSI ≥12 were classified as severe PARDS, and children with OSI <12 were classified as mild/moderate PARDS. OSI was obtained adjusting for altitude and using instances with SpO_2_ values ≤97%. Outcomes included maximum PELOD-2 and nonpulmonary PELOD-2 score (without respiratory component) during the first week of invasive mechanical ventilation, death before PICU discharge, duration of invasive mechanical ventilation, 28-day ventilator-free days (VFDs), and PICU length of stay (LOS). Liberation from invasive mechanical ventilation >48 h defined successful extubation.

### Molecular Measurements

**Figure 1.**
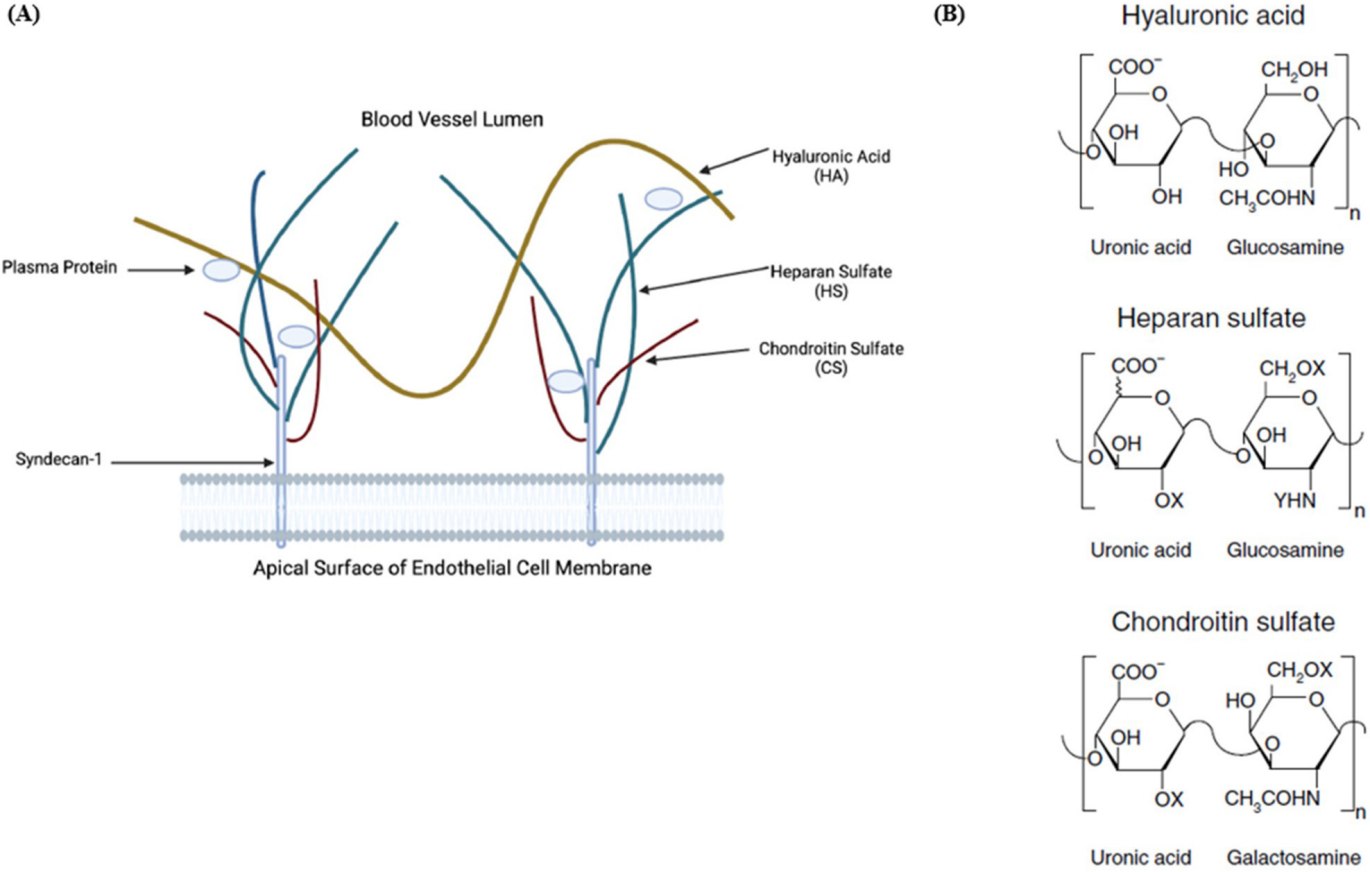
(**A**) Graphical representation of the pulmonary vascular endothelial glycocalyx. The main constituents include proteoglycans (eg, syndecan-1), glycosaminoglycans (eg, HS, CS, and HA), integrating plasma proteins, and glycoproteins (not pictured) HA, unlike other glycosaminoglycans, is not covalently linked to proteoglycans, but can be noncovalently bound to cell-surface glycoproteins (not pictured). (**B**) Glycosaminoglycan disaccharide structure. Glycosaminoglycans are composed of long chains of repeating disaccharide units of a hexuronic acid (eg, GlcA or IdoA linked to an amino sugar (eg, GlcNAc or GalNAc). HS and CS can additionally be modified by sulfation at the X or Y positions (X = SO_3_H or H; Y = SO_3_H or COCH_3_). n = number of disaccharide unit repeats. Figures were created with biorender.com and were not drawn to scale. Abbreviations: CS, chondroitin sulfate; HA, hyaluronic acid; HS, heparan sulfate; GlcA, glucuronic acid; IdoA, iduronic acid;GalNAc, galactosamine; GlcNAc, glucosamine. Created with BioRender.com.

The blood was collected in an ethylenediaminetetraacetic acid (EDTA) tube, placed immediately on ice after collection, and centrifuged at 2500*g* for 10 min at 4°C. The plasma was aliquoted and stored at −80°C. We employed liquid chromatography-tandem mass spectrometry multiple reaction monitoring to characterize and quantify GAG class (heparan sulfate [HS], chondroitin sulfate [CS], and hyaluronan [or hyaluronic acid, HA], and disaccharide sulfation subtype [HS and CS] [Figure 1]). GAGs were isolated from EDTA plasma using a spin column approach as has been previously described and validated.^29-31^ After desalting, the GAGs were enzymatically digested into component disaccharides. The GAGs were then labeled with 2-aminoacridone (AMAC). Taking these AMAC-labeled disaccharides, GAG class and their respective sulfation subtypes were separated and quantified using mass spectrometry. Plasma syndecan-1, an abundant endothelial-derived HS and CS PG found within the microvascular EGCX, was assayed using an enzyme-linked immunoassay (ab46506, Abcam, Cambridge MA, USA). Standard curves had an R^2^ of >95%, and assays were performed in duplicate with an intra-assay coefficient of variation <10%. The final recorded value was the average of the two assays.

### Statistical Analysis

Categorical variables were presented as frequency and proportion (%) and were compared using Chi-squared test (χ^2^) or Fisher's exact test, as appropriate. EGCX biomarker levels (GAGs and syndecan-1) were non-normally distributed continuous variables, and raw values were presented as median and interquartile range (IQR) with group differences assessed using Wilcoxon rank-sum test. GAG sulfation subtypes were evaluated as mean proportions of total HS and CS (expressed as % ± SD) and compared using unequal variances *t*-test. For EGCX biomarker levels, the association between EGCX biomarker quartile and PARDS presence and severity was assessed by Cochran-Armitage test for trend. EGCX biomarkers, maximum PELOD-2, and nonpulmonary PELOD-2 scores were log_10_-transformed and correlations assessed by Pearson's correlation coefficients (*r*) among children with sepsis-associated PARDS. Univariate and multivariable linear regression (coefficient [β]) tested the associations between log_10_-transformed EGCX biomarkers and VFDs and PICU LOS in children with sepsis-associated PARDS. Models were adjusted for age, sex, PRISM-III, and immunocompromised status. Covariates were chosen based upon known associations with these variables and PARDS clinical outcomes.^32,33^ All analyses included 95% confidence intervals and two-tailed *p*-values based on robust standard error estimates with a significance level of <0.05. As this was an exploratory analysis, we did not correct for multiple comparisons. Finally, we performed *post hoc* sensitivity analyses removing immunocompromised patients from the group comparisons (children with vs without sepsis-associated PARDS) to address bias potentially introduced by the presence of this group exclusively among the PARDS patients. Analyses were performed using STATA SE version 17 (StataCorp, College Station, TX).

## Results

### Clinical Characteristics

We evaluated 39 children: 29 mechanically ventilated with sepsis-associated PARDS and 10 mechanically ventilated without PARDS (Supplemental Figure 1). Demographic characteristics, illness severity, and patient outcomes were similar between groups (Table 1). Among patients with sepsis-associated PARDS, PARDS diagnosis occurred shortly after intubation (median 0.02 days [IQR 0.01-0.12]). Nonpulmonary sepsis was the etiology of lung injury in 28% (n = 8/29) of sepsis-associated PARDS patients. Plasma collection occurred a similar number of days after initiation of invasive mechanical ventilation in patients with and without PARDS (median 4.5 days [IQR 3.4-5.6] and 4.3 [IQR 3.1-5.3], respectively) (Table 1).

**Table 1.** Patient Demographics and Clinical Characteristics by Group.

Patient characteristics and outcomes	Sepsis-associated PARDS (n = 29)	Mechanically ventilated without PARDS (n = 10)	*p^d^*
**Demographics**			
Age, year	6.4 (1.1-12.2)	6.7 (1.9-10.4)	0.92
Weight, kg	20.9 (8.0-39.8)	26.6 (13.0-38.0)	0.93
Female sex	15 (52)	4 (40)	0.52
**Comorbidities**			
Chronic pulmonary disease	9 (31)	2 (20)	0.50
Neurological disease	9 (31)	2 (20)	.50
Immunocompromised^a^	6 (21)	0 (0)	0.30
**Illness severity**			
Oxygen saturation index^b^	9.6 (5.8-13.1)	1.9 (1.8-4.4)	**<0**.**01**
PRISM-III	4.0 (0.0-9.0)	5.5 (0.0-11.0)	0.85
**Timing of plasma acquisition**			
Days from *ntubation*	4.5 (3.4-5.6)	4.3 (3.1-5.3)	0.66
**PARDS cause**			
Nonpulmonary sepsis	8 (28)	NA	
Pulmonary sepsis	21 (72)		
**PARDS severity**			
Mild/moderate	20 (69)	NA	
Severe	9 (31)		
**Outcomes**			
PELOD-2^b^	8.4 (2.2-19.1)	10.7 (2.2-12.9)	0.78
Nonpulmonary PELOD-2^b^	1.4 (0.5-3.5)	2.8 (0.55-3.5)	0.82
Duration of invasive ventilation, days	6.8 (5.7-10.8)	7.0 (5.6-8.8)	0.56
28-Day Ventilator-free days^c^	20.8 (16.2-22.3)	21.0 (19.1-22.4)	0.44
PICU LOS, days	12.8 (8.6-15.4)	14.3 (6.5-15.8)	0.63
PICU survivors	28 (97)	10 (100)	1.00

Data are presented as median (interquartile range) or n (%).

^a^ Immunocompromised included patients with oncological diagnoses or were post-hematopoietic cell transplantation.

^b^ Oxygen saturation index, PELOD-2, and nonpulmonary PELOD-2 represent maximum values (highest values during the first week of invasive mechanical ventilation).

^c^ Ventilator-free days censored at 28 days post intubation.

^d^ Group comparisons were performed using the Wilcoxon rank-sum test, χ^2^ test, or Fisher's exact test, as appropriate.

*p-*value in bold reflects statistically significant difference (*p <*0.05).

Abbreviations: PICU LOS, pediatric intensive care unit length of stay; PARDS, pediatric acute respiratory distress syndrome; PELOD-2, Pediatric Logistic Organ Dysfunction-2 Score; PRISM-III, Pediatric Risk of Mortality-III score.

### Glycosaminoglycans

**Figure 2.**
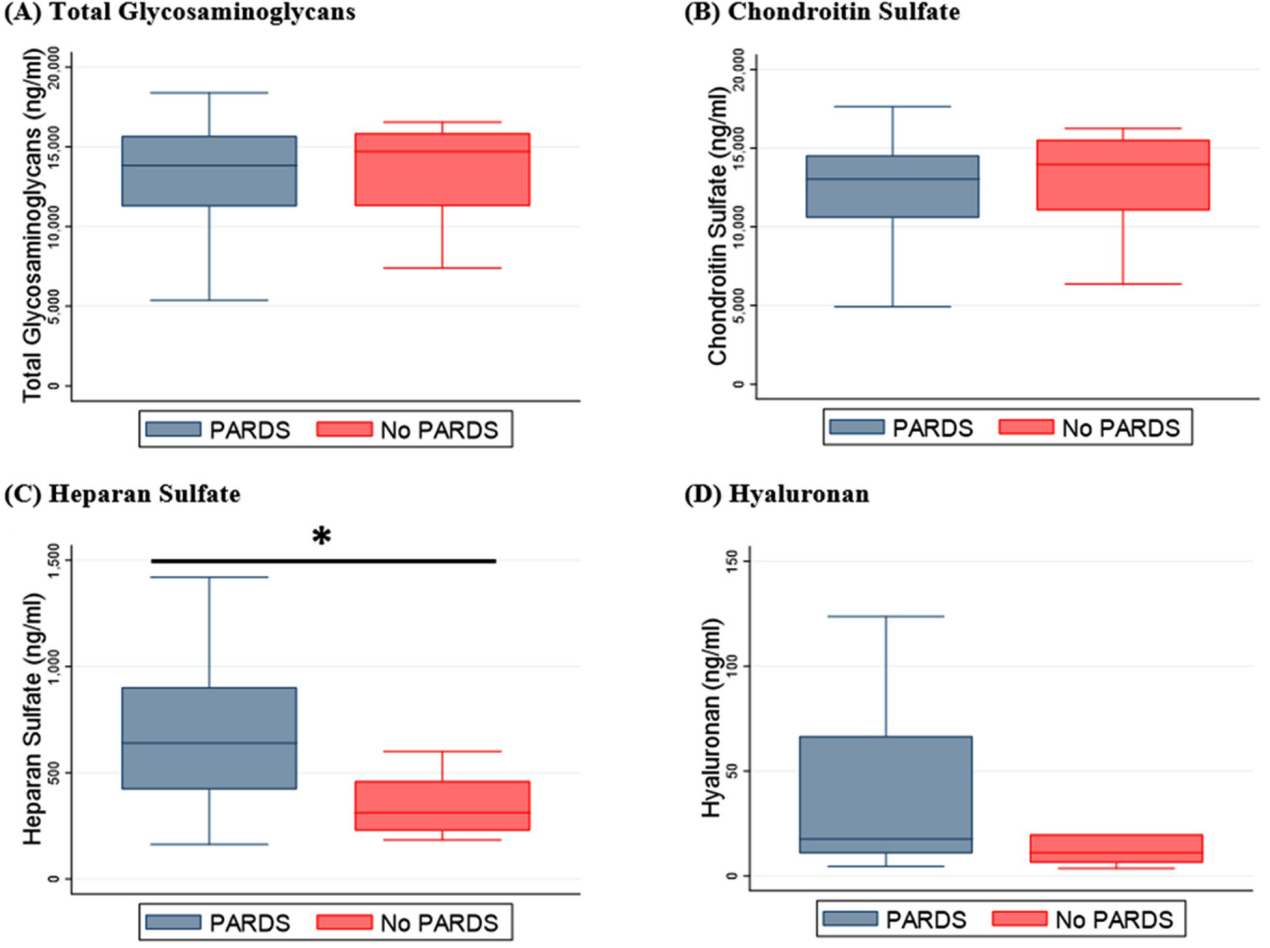
Plasma concentrations of total glycosaminoglycans (**A**), chondroitin sulfate (**B**), heparan sulfate (**C**), and hyaluronan (**D**) among children with sepsis-associated PARDS (n = 29) compared to mechanically ventilated children without PARDS (n = 10). Group comparisons were performed using the Wilcoxon rank-sum test. *p-value <0.05. Abbreviation: PARDS, pediatric acute respiratory distress syndrome.

Plasma HS concentration was significantly higher in patients with sepsis-associated PARDS (median 639 ng/mL [IQR 421-902] vs 311 [IQR 228-461], *p *= 0.01) compared to patients mechanically ventilated without PARDS (Figure 2C; Supplemental Table 1). Total GAGs and CS levels did not vary between groups; HA levels were higher in sepsis-associated PARDS, but these differences were not statistically significant (Figure 2A, B, and D, Supplemental Table 1). The highest two quartiles of HS levels contained 62% of the sepsis-associated PARDS patients compared to 80% of patients without PARDS occupying the lowest two quartiles (*p *=0.01 for test of trend, Supplemental Figure 2A).

Immunocompromised children were exclusively represented in the sepsis-associated PARDS group (n = 6). Immunocompromised children with PARDS had significantly higher concentrations of HS relative to children without PARDS (median 758 [IQR 609-902] vs 311 [IQR 228-461], *p *< 0.01). After removing immunocompromised patients from the analyses, HS remained significantly higher in children with compared to without PARDS (*p *= 0.03). CS and HA levels remained similar between groups.

### Glycosaminoglycan Ultrastructural Characterization and Quantification

**Figure 3.**
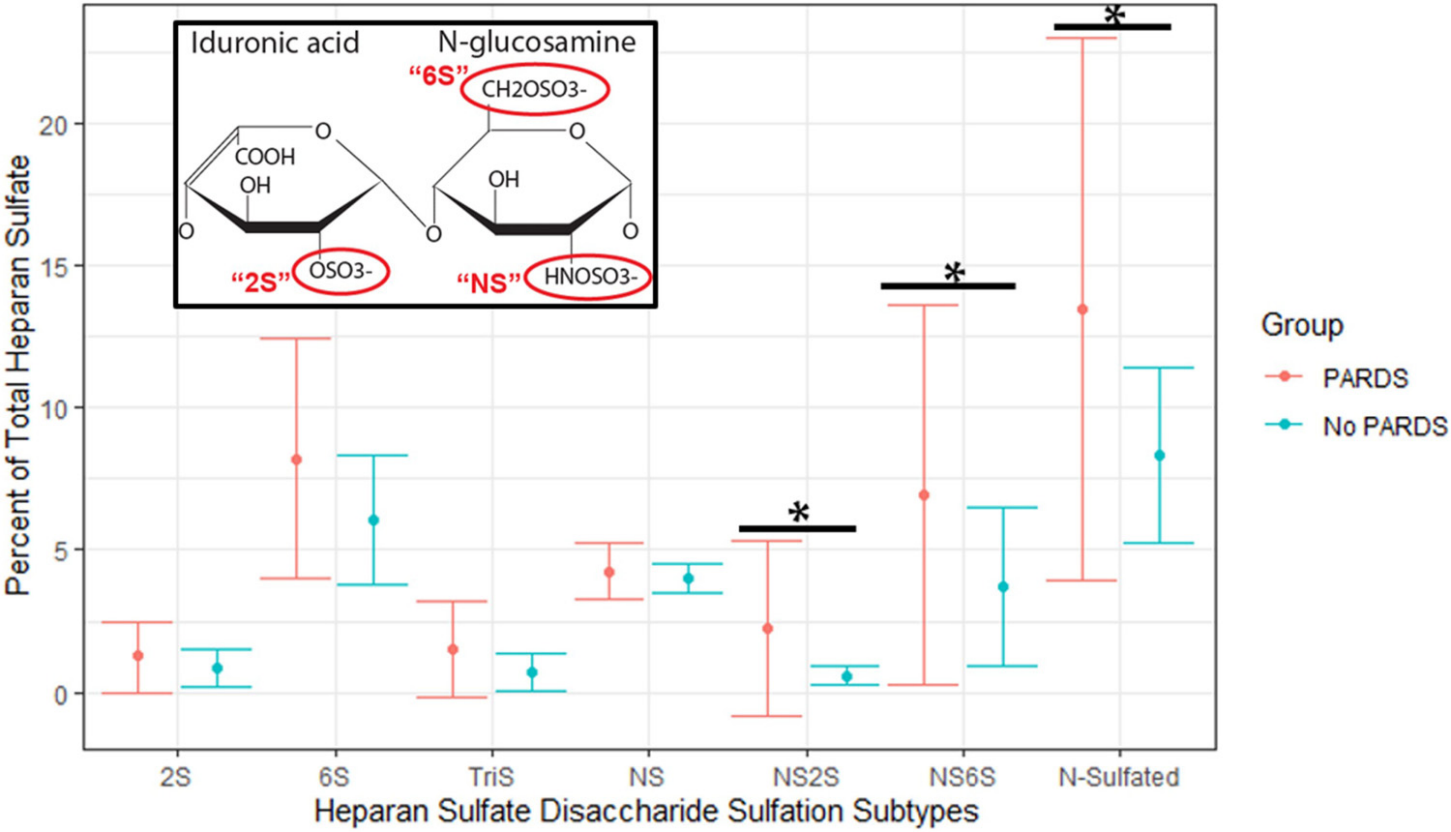
HS disaccharide sulfation subtype analysis. HS disaccharide structure illustrated with respective sulfation modifications: HS can be modified with sulfo-groups at the 2-O position (2S) of its uronic acid residue (IdoA featured here) and/or the N-acetyl (NS) and 6-O (6S) positions of its GlcNAc residue. Values presented reflect proportions (%, ± SD) as a function of total HS. NS2S, NS6S, *N*-sulfated (NS2S + NS6S + NS) subtypes represented greater proportions of total HS in children with sepsis-associated PARDS relative to mechanically ventilated children without PARDS (*all *p *< 0.05). 0S sulfation subtypes (HS disaccharides without sulfation) were significantly lower in PARDS (*p *< 0.05) (data not shown). Note: 2S6S HS subtype was not detectable in either group. Abbreviations: Heparan sulfate, HS; GlcNAc, glucosamine; IdoA, iduronic acid; PARDS, pediatric acute respiratory distress syndrome; SD, standard deviation.

Comparison of HS disaccharide sulfation subtypes demonstrated that the NS2S (2.3% [SD ±3.1] vs 0.6% [SD ±0.3], *p *< 0.01) and NS6S (7.0% [SD ±6.6] vs 3.7% [SD ±2.7], *p = *0.03) proportions were higher in children with sepsis-associated PARDS relative to mechanically ventilated children without PARDS (Figure 3). 0S sulfation subtypes (HS disaccharides without sulfation) were lower in PARDS (75.5% [SD ±15.4] vs 84.2% [SD ±6.5], *p = *0.02). *N*-sulfated HS disaccharides (NS2S + NS6S + NS) represented 13.4% (SD ±9.5) of total HS in children with sepsis-associated PARDS compared to 8.3% (SD ±3.1) among mechanically ventilated children without PARDS (*p *= 0.01). *N-*sulfated HS levels by quartile were associated with the presence of sepsis-associated PARDS (*p *< 0.01 for test for trend, Supplemental Figure 2B). Except for the 2S4S CS subtype (*p *= 0.02), no other CS subtype level was significantly higher in sepsis-associated PARDS (data not shown).

### Glycosaminoglycans and Clinical Outcomes

**Figure 4.**
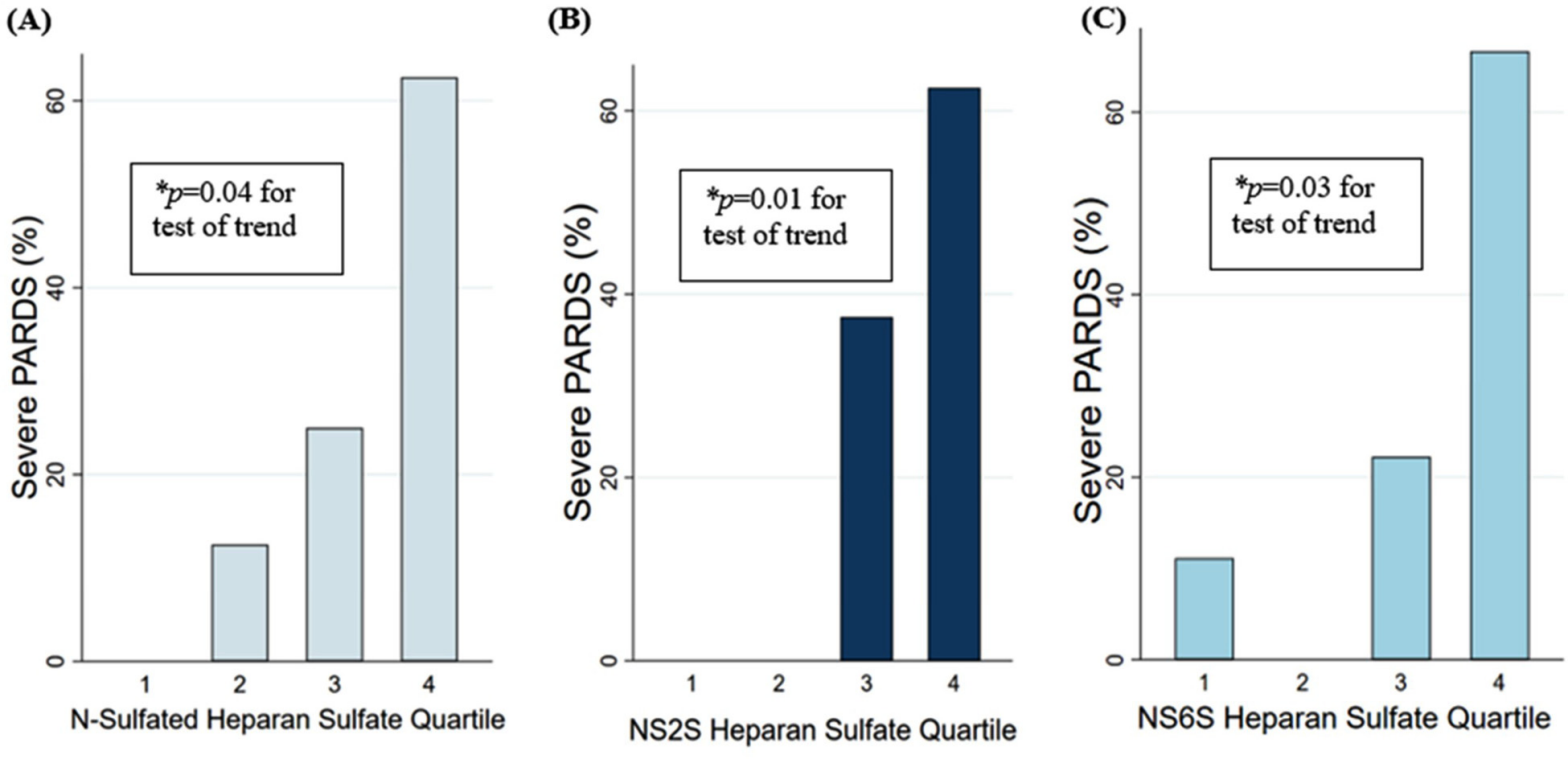
Increasing plasma concentrations by quartile of (**A**) *N*-sulfated (**B**) NS2S and (**C**) NS6S HS disaccharides were associated with higher proportions of severe sepsis-associated PARDS patients (Cochran-Armitage test for trend). Abbreviations: HS, heparan sulfate; PARDS, pediatric acute respiratory distress syndrome.

We tested the associations between plasma GAG levels and clinical outcomes among children with sepsis-associated PARDS. Plasma HS concentration was significantly higher in severe PARDS (n = 9/29) relative to mild/moderate PARDS (median 824 [IQR 717-1129] vs 583 [IQR 328-773], *p = *0.04). HA and CS did not significantly vary between severe and mild/moderate PARDS. Although each of the HS disaccharide subtype levels were higher, only the NS6S subtype was significantly higher in severe PARDS (compared to mild/moderate PARDS) (*p = *0.04, Supplemental Table 2). The highest quartile of HS levels contained 56% of the severe PARDS patients compared to 0% in the lowest HS quartile (*p = *0.05 for test for trend). Higher concentrations of NS2S, NS6S, and *N-*sulfated subtypes (NS2S + NS6S + NS) by quartile were associated with severe PARDS (all *p *< 0.05 for test for trend). The highest quartile of these HS sulfation subtypes contained >60% of severe PARDS patients (Figure 4A-C**).** In comparison, only 20% to 25% of mild/moderate PARDS patients occupied the highest quartile of HS and the respective HS sulfation subtypes.

Among children with sepsis-associated PARDS, median 28-day VFDs were 20.8 (IQR 16.2-22.3) with three patients assigned VFDs of 0. After adjusting for sex, age, PRISM-III, and immunocompromised status, higher plasma levels of HS and HS sulfation subtypes were associated with fewer VFDs, except the 0S and 2S subtypes (all *p *< 0.05) (Supplemental Table 3). Total HS and HS disaccharide subtypes were not associated with PELOD-2, nonpulmonary PELOD-2, and PICU LOS (Supplemental Table 3).

### Proteoglycans

Plasma syndecan-1 concentration was significantly higher in children with sepsis-associated PARDS (median 146 ng/mL [IQR 32-315] vs 8 [IQR 8-50], *p = *0.01) compared with patients without PARDS. After removal of immunocompromised patients, syndecan-1 remained associated with sepsis-associated PARDS (*p = *0.04). Among children with sepsis-associated PARDS, syndecan-1 concentrations positively correlated with total HS (*r *= 0.63, *p *< 0.01), but not total CS (*r *= 0.21, *p *= 0.26) (Supplemental Figure 3). Syndecan-1 levels were positively correlated with PELOD-2 scores, but were not associated with VFDs or PICU LOS in unadjusted or adjusted analyses (Supplemental Table 3).

## Discussion

We identified that circulating HS disaccharides and syndecan-1 levels were higher in mechanically ventilated children with sepsis-associated PARDS relative to children without PARDS. Furthermore, among children with PARDS, greater degrees of HS disaccharide fragmentation were associated with worse lung injury and independently associated with fewer VFDs. Our findings suggest that sepsis-associated PARDS pathobiology may be in part characterized by EGCX degradation and, in our study, more severe EGCX degradation was associated with adverse outcomes.

The EGCX is increasingly recognized as a gatekeeper of vascular homeostasis. It forms an endothelial surface layer critical to regulating vascular permeability, mechanosignaling, and leukocyte adhesion.^34^ Degradation of the EGCX during sepsis via linked mechanisms of GAG fragmentation and PG cleavage promotes increased vascular permeability, impaired vasoreactivity, and enhanced inflammation. The clinical manifestations and severity of these processes, however, varies depending on the organ affected. Recent advancements in glycoanalytical methods have provided evidence that the structure of the EGCX varies depending on the organ and microvascular bed in which it is located.^13^ Therefore, delineating the ultrastructural characteristics of the EGCX may be important in unraveling its functional role in PARDS pathobiology.

Our study utilized a state-of-the-art mass spectrometry technique to enable the ultrastructural characterization and quantification of plasma GAGs in children mechanically ventilated for acute respiratory failure. Children with sepsis-associated PARDS exhibited prominent fragmentation of highly sulfated HS disaccharides, suggesting their potential as biomarkers for pulmonary microvascular endothelial dysfunction. In a seminal study by Schmidt et al,^16^ endotoxemia in mice caused pulmonary microvascular EGCX degradation via tumor necrosis factor-α mediated induction of endothelial heparanase (a HS-specific glucuronidase). Heparanase potentiated the specific fragmentation of highly sulfated HS disaccharides into circulation causing endothelial barrier dysfunction, neutrophil adhesion, and acute lung injury.^18,35^ Moreover, a revision of the classic Starling principle ascribes an essential role of the EGCX in regulating transvascular fluid and protein flux.^36^ Under this revised equation, the plasma and subglycocalyx colloid osmotic pressure difference is a principal determinant of transendothelial flow.^16^ Inflammatory conditions that degrade the EGCX promote paracellular movement of plasma fluid and protein, facilitating the development of tissue edema characteristic of acute lung injury.^17,23^ While the precise ultrastructural contribution of the pulmonary EGCX (and concurrently, HS) to endothelial permeability is debated, it serves a major role in regulating mechanosignaling pathways that impact endothelial barrier function.^14,37^

There are few clinical studies that have specifically and simultaneously measured PGs (syndecan-1) and the ultrastructural characteristics of GAGs (HS, HA, and CS) in adult or pediatric ARDS. In a small cohort of adults intubated for respiratory failure, plasma HS concentrations (particularly highly sulfated HS disaccharides) were higher among patients with nonpulmonary sepsis (compared to healthy controls).^38^ Preclinical data has demonstrated heparanase inhibitors attenuate lipopolysaccharide-induced HS fragmentation, endothelial hyperpermeability, and neutrophil extravasation.^18^ These data as well as the concordance between endothelial-derived syndecan-1 and HS in our study, supports plasma HS as a potent index of EGCX degradation. We similarly observed higher circulating levels of syndecan-1 in sepsis-associated PARDS patients. Preclinical data suggest that during sepsis, syndecan-1 ectodomains can be cleaved from the endothelial cell membrane by matrix metalloproteinases (MMPs).^39-42^ The precise interplay between heparanase-mediated HS disaccharide fragmentation and MMP-mediated syndecan-1 cleavage is evolving, with evidence demonstrating that heparanase plays a role in enhancing syndecan-1 shedding by upregulating MMP expression.^43^

Disruption of the EGCX not only creates a vulnerable endothelial surface layer potentiating acute lung injury, but circulating fragments act as systemic effectors capable of propagating systemic organ dysfunction.^22^ Specifically, HS disaccharides can function as damage-associated-molecular patterns by binding toll-like receptor 4, augmenting inflammatory signaling.^21,44^ While our study suggests a unique role for HS and its highly sulfated subtypes in PARDS pathobiology (and less so HA or CS), future investigations should further elucidate the relationship between HS disaccharides and systemic organ dysfunction. Additionally, variations in our findings with others may reflect differences in study methodology or the compartment with which these biomarkers were measured (eg, urine, airway, and plasma). For example, in other adult ARDS cohorts, CS and HA levels have been more strongly associated with severity of lung injury, organ dysfunction, and in-hospital mortality.^30,45^ Accordingly, further study is needed to define EGCX degradation signatures in different biological compartments offering further insights in PARDS pathobiological heterogeneity.

The kinetics of EGCX degradation and clearance are important to consider as the time of plasma collection was approximately four days after initiation of invasive mechanical ventilation. Preclinical studies suggest that HS fragments are rapidly cleared from the plasma of septic animals (t_1/2_ ∼90 min), and the persistence of HS in plasma could reflect ongoing EGCX degradation.^44^ Indeed, sepsis mediators can continuously degrade the EGCX for up to a week after sepsis onset and impact the EGCX's reconstitution, and accordingly, contribute to lung injury severity and impaired resolution.^46,47^ We found that greater degrees of HS disaccharide fragmentation were associated with worse lung injury and fewer VFDs. The highest quartile of HS and its highly sulfated subtypes contained between 56% and 67% of the severe PARDS patients, with HS disaccharides maintaining their association with VFDs even after adjustment for potential confounders. Although we cannot definitively exclude impaired clearance, we suspect that the associations observed better reflect persistent EGCX degradation. Future studies should consider multiple time points of plasma collection to outline a detailed signature of EGCX degradation in PARDS.

Our study has important limitations. First, this investigation was a small, single center, observational study, which limits its generalizability. Additionally, as this was a secondary analysis of a prospective cohort, sample collection times were not designed to answer the specific hypotheses proposed in this analysis. Accordingly, the differences in EGCX degradation biomarkers observed between our groups argue for more robust studies to establish EGCX degradation products as reliable biomarkers of endothelial dysfunction capable of discriminating PARDS from other pathologies and risk stratifying outcomes. Second, a convenience sample of children with sepsis-associated PARDS and mechanically ventilated comparators without PARDS were selected from a larger parent cohort raising the potential for selection bias. We mitigated this by detailed classification of the patient groups by two observers (CJS and ABM) blinded to mass spectrometry data. We are reassured that our comparator group was critically ill, mechanically ventilated, and differed clinically by absence of lung injury and sepsis. In future studies, a broader evaluation across sepsis cohorts, inclusive of noninvasively ventilated patients, could enrich our understanding of the extent to which the EGCX is degraded along the continuum of sepsis-associated organ injury. Third, there is risk for residual confounding. Various factors, including fluid management, mechanical ventilation strategies, and adjunctive therapies, may have influenced the observed relationships. Despite being a mainstay of sepsis management, fluid resuscitation may paradoxically worsen organ dysfunction by degrading the EGCX.^31,48^ Established data in adult and pediatric ARDS consistently show that excess fluid accumulation correlates with poor outcomes such as compromised oxygenation, fewer VFDs, and increased mortality.^49-51^ Contemporary PARDS cohorts should investigate injudicious fluid management's mechanistic contributions to outcomes mediated through EGCX destruction. However, our associations were maintained even after adjustment for confounders and removal of immunocompromised patients, a group that when suffering from PARDS may be associated with a higher susceptibility to and degree of endothelial injury.^52,53^

Taken together, these results suggest that EGCX degradation as reflected by increased concentrations of highly sulfated HS disaccharides and syndecan-1 may be in part associated with the sepsis-associated PARDS pathobiology. There remains an incomplete understanding of the key inflection points that mediate pulmonary microvascular endothelial dysfunction supporting an ongoing need to study the mechanistic role of EGCX degradation in children with PARDS. The development of therapeutic strategies aimed at endothelial preservation and restoration, where mitigating EGCX injury and facilitating its repair, could impact PARDS clinical outcomes. Studies into the early detection of EGCX disruption may allow for prompt intervention before the clinically apparent PARDS phenotype manifests. This could inform treatment strategies regarding fluid management, corticosteroids, and anticoagulation, all therapies with the potential to attenuate EGCX destruction or restore function.^54-56^ Accordingly, future derivation and validation of biologically distinct patient subgroups characterized by patterns of EGCX degradation may allow for advancement of precision medicine approaches where patients are matched to treatments guided by underlying glycobiology.

## Conclusions

In conclusion, our study evaluated plasma EGCX degradation biomarkers in mechanically ventilated children with and without sepsis-associated PARDS, and the associations between EGCX degradation biomarkers and PARDS clinical outcomes. Circulating HS disaccharides and syndecan-1 were increased in sepsis-associated PARDS relative to children without PARDS. Among children with sepsis-associated PARDS, HS disaccharides were associated with worse lung injury and fewer VFDs. By applying state-of-the-art mass spectrometry analyses, this study represents a first-step in characterizing novel EGCX degradation biomarkers and providing potential insights into the connection between EGCX degradation, pulmonary microvascular endothelial dysfunction, and PARDS pathobiology. Larger cohorts are needed to validate these findings, test EGCX degradation biomarkers prognostic and predictive significance in PARDS, and determine their potential candidacy for targeted therapeutic intervention.

## Supplemental Material

sj-docx-1-jic-10.1177_08850666231200162 - Supplemental material for Endothelial Glycocalyx Degradation Patterns in Sepsis-Associated Pediatric Acute Respiratory Distress Syndrome: A Single Center Retrospective Observational Study Click here for additional data file. Supplemental material, sj-docx-1-jic-10.1177_08850666231200162 for Endothelial Glycocalyx Degradation Patterns in Sepsis-Associated Pediatric Acute Respiratory Distress Syndrome: A Single Center Retrospective Observational Study by Colin J. Sallee, Joseph A. Hippensteel, Kristen R. Miller, Kaori Oshima, Andrew T. Pham, Robert P. Richter, John Belperio, Yamila L. Sierra, Andreas Schwingshackl, Peter M. Mourani, Eric P. Schmidt, Anil Sapru and Aline B. Maddux in Journal of Intensive Care Medicine
