# Supplementary material for: Endothelial Glycocalyx Degradation Patterns in Sepsis-Associated Pediatric Acute Respiratory Distress Syndrome: A Single Center Retrospective Observational Study
Source: J Intensive Care Med. 2023 Sep 6;39(3):277–87. doi: 10.1177/08850666231200162 (PMC10845819; doi:10.1177/08850666231200162)
Supplement: sj-docx-1-jic-10.1177_08850666231200162 - Supplemental material for Endothelial Glycocalyx Degradation Patterns in Sepsis-Associated Pediatric Acute Respiratory Distress Syndrome: A Single Center Retrospective Observational Study [file sj-docx-1-jic-10.1177_08850666231200162.docx]

**Endothelial Glycocalyx Degradation Patterns in**

**Sepsis-Associated Pediatric Acute Respiratory Distress Syndrome:**

**A Single Center Retrospective Observational Study**

**Supplemental Content**

**Colin J. Sallee, MD, MS^1^**, Joseph A. Hippensteel, MD^2^, Kristen R. Miller, MS^3^, Kaori Oshima, PhD^4^, Andrew T. Pham, MD^2^, Robert P. Richter, MD^5^, John Belperio, MD^6^, Yamila L. Sierra, MPH^3^, Andreas Schwingshackl, MD, PhD^1^, Peter M. Mourani, MD^7^, Eric P. Schmidt, MD^4^, Anil Sapru, MD, MAS^1^, Aline B. Maddux, MD, MSCS^3^

^1^Department of Pediatrics, Division of Pediatric Critical Care Medicine, David Geffen School of Medicine at University of California Los Angeles and Mattel Children's Hospital, Los Angeles, CA, United States

^2^Department of Medicine, Division of Pulmonary Sciences and Critical Care Medicine, University of Colorado Anschutz Medical Campus, Aurora, CO, United States

^3^Department of Pediatrics, Section of Pediatric Critical Care, University of Colorado School of Medicine and Children's Hospital Colorado, Aurora, CO, United States

^4^Department of Medicine, Division of Pulmonary and Critical Care Medicine, Harvard Medical School and Massachusetts General Hospital, Boston, MA, United States,

^5^Department of Pediatrics, Division of Pediatric Critical Care Medicine, University of Alabama at Birmingham Heersink School of Medicine, Birmingham, AL, United States,

^6^Department of Medicine, Division of Pulmonary Critical Care and Sleep Medicine, David Geffen School of Medicine at University of California Los Angeles and Ronald Reagan Medical Center, Los Angeles, CA, United States

^7^Department of Pediatrics, Division of Pediatric Critical Care Medicine, University of Arkansas for Medical Sciences and Arkansas Children’s Hospital, Little Rock, AR, United States

**Table of Contents (in order of appearance in manuscript)**:

Supplemental Figure 1. Enrollment Diagram

Supplemental Table 1. Glycosaminoglycan Concentrations by Group

Supplemental Figure 2. Association between Heparan Sulfate and *N*-sulfated Heparan Sulfate Concentration by Quartile and Sepsis-Associated PARDS

Supplemental Table 2. Heparan Sulfate Disaccharide Sulfation Subtypes by PARDS Severity

Supplemental Table 3. Relationship between Endothelial Glycocalyx Biomarkers and Outcomes in Sepsis-Associated PARDS

Supplemental Figure 3. Correlation of Syndecan-1 with Heparan Sulfate and Chondroitin Sulfate

**Parent Study:**

**137** children receiving

invasive mechanical ventilation ≥ 72 hours

**79** excluded:

Did not consent for blood draws (n=58)

Consented for blood draw but specimen unable to be collected (n=17)

Withdrawn due to inappropriate enrollment (n=2)

Unable to complete assent (n=1)

Specimen insufficient for GAG analysis (n=1)

**58** screened for inclusion

**21** without PARDS

**37** with PARDS

**11** excluded:

Septic shock (n=2)

Unspecified lung disease (n=4)

Trauma (n=3)

Other (n=2)

**8** excluded:

Transfusion (n=1)

Other/Unspecified (n=5)

Aspiration (n=1)

Drowning (n=1)

**29 with PARDS mechanically ventilated with pulmonary or nonpulmonary sepsis**

**10 without PARDS mechanically ventilated for neurological failure or nonpulmonary procedure/surgery**

**Supplemental Figure 1.** Enrollment diagram. Children with sepsis-associated pediatric acute respiratory distress syndrome (PARDS) were compared to children without PARDS receiving invasive mechanical ventilation for neurological failure (e.g., inability to protect airway) or nonpulmonary procedure/surgery. Sepsis-associated PARDS represented children with a known or suspected source of infection from a pulmonary or nonpulmonary source. For the comparator group mechanically ventilated without PARDS, children with sepsis or other potential sources of lung injury were excluded.

**Supplemental Table 1**. **Glycosaminoglycan Concentrations by Group**

| **GAG Class^a^** | **Sepsis-Associated PARDS**  **(n=29)** | **Mechanically Ventilated**  **without PARDS**  **(n=10)** | ***p^b^*** |
| --- | --- | --- | --- |
| Total | 13842  (11254-15688) | 14722  (11287-15866) | 0.77 |
| Chondroitin sulfate | 13026  (10587-14547) | 13969  (11040-15532) | 0.74 |
| Heparan sulfate | 639  (421-902) | 311  (228-461) | **0.01** |
| Hyaluronan | 18  (11-67) | 11  (6-20) | 0.14 |

GAG = glycosaminoglycan; PARDS = pediatric acute respiratory distress syndrome.

^a^ GAG concentration (ng/ml) with median (interquartile range)

^b^ Group comparisons were performed using the Wilcoxon rank-sum test. *p-*value in bold reflects statistically significant difference (*p<*0.05).

(**A**)

**p*=0.01 for test of trend

(**B**)

**p*<0.01 for test of trend

**Supplemental Figure 2.** (**A**) Higher plasma heparan sulfate (HS) levels by quartile were associated with sepsis-associated pediatric acute respiratory distress syndrome (PARDS). (**B**) Higher plasma *N*-sulfated HS disaccharides by quartile (which includes NS2S, NS6S, and NS subtypes) were associated with sepsis-associated PARDS.

**Supplemental Table 2. Heparan Sulfate Disaccharide Sulfation Subtypes by PARDS Severity**

| **Heparan Sulfate Disaccharides^a^** | **Severe PARDS** | **Mild/Moderate PARDS** | ***p^b^*** |
| --- | --- | --- | --- |
| Total HS | 824 (717-1129) | 583 (328-772) | **0.04** |
| TriS | 14.2 (7.3-33.4) | 4.1 (1.5-14.6) | 0.17 |
| NS2S | 14.6 (5.7-40.3) | 4.8 (1.9-13.2) | 0.08 |
| NS6S | 82.2 (30.8-131.2) | 17.1 (11.1-52.1) | **0.04** |
| NS | 26.9 (20.8-50.0) | 23.5 (12.5-39.7) | 0.19 |
| 0S | 714 (386-751) | 454 (279-590) | 0.11 |
| 2S | 8.4 (5.0-18.6) | 3.8 (1.7-8.8) | 0.06 |
| 6S | 65.3 (33.4-152.5) | 33.1 (16.6-76.5) | 0.13 |
| *N*-Sulfated | 128.8 (70.3-322.5) | 46.0 (24.6-110.7) | 0.06 |

PARDS = pediatric acute respiratory distress syndrome; *N*-sulfated heparan sulfate = NS2S + NS6S + NS

^a^ GAG concentration (ng/ml) with median (interquartile range)

^b^ Group comparisons were performed using the Wilcoxon rank-sum test. *p-*value in bold reflects statistically significant difference (*p<*0.05).

**Supplemental Table 3. Relationship between Endothelial Glycocalyx Biomarkers and Outcomes in Sepsis-Associated PARDS**

| **EGCX Biomarkers** | **PELOD-2^b^** | **Nonpulmonary PELOD-2^b^** | **Unadjusted 28-Day VFDs^c^** | **Adjusted 28-Day VFDs^c,d^** | **Unadjusted PICU LOS^c^** | **Adjusted PICU LOS^c,d^** |
| --- | --- | --- | --- | --- | --- | --- |
| **GAG Class^a^** |  |  |  |  |  |  |
| Chondroitin  sulfate | *r*=0.13  *p=*0.51 | *r*=0.16  *p=*0.40 | **-6.2 (-11.3, -1.1)**  ***p=*0.03** | **-7.1 (-12.6, -1.7)**  ***p=*0.01** | 1.1 (-3.1, 5.1)  *p*=0.60 | 1.4 (-3.1, 5.9)  *p*=0.53 |
| Heparan  sulfate | *r*=0.29  *p=*0.13 | *r*=0.24  *p=*0.20 | **-3.5 (-6.6, -0.34)**  ***p*=0.03** | **-3.6 (-7.2, -0.10)**  ***p*=0.04** | 0.4 (-2.5, 3.3)  *p*=0.76 | 1.5 (-1.3, 4.3)  *p*=0.30 |
| Hyaluronan | *r*=0.23  *p=*0.22 | *r*=0.22  *p=*0.25 | -1.4 (-3.2, 0.36)  *p*=0.11 | -1.6 (-3.4, 0.22)  *p*=0.08 | 0.29 (-1.1, 1.7)  *p*=0.68 | 0.88 (-0.42, 2.2)  *p*=0.18 |
| **HS Disaccharide**  **Sulfation Subtype^a^** |  |  |  |  |  |  |
| TriS | *r*=0.08  *p=*0.68 | *r*=0.06  *p=*0.75 | **-2.1 (-3.9, -0.28)**  ***p*=0.02** | **-2.2 (-4.2, -0.26)**  ***p*=0.03** | 0.29 (-1.4, 2.0)  *p*=0.73 | 0.52 (-1.3, 2.4)  *p*=0.57 |
| NS2S | *r*=0.19  *p=*0.33 | *r*=0.15  *p=*0.44 | **-1.8 (-3.4, -0.28)**  ***p*=0.02** | **-1.9 (-3.9, -0.34)**  ***p*=0.02** | 0.29 (-1.1, 1.6)  *p*=0.66 | 0.67 (-0.73, 2.1)  *p*=0.34 |
| NS6S | *r*=0.17  *p=*0.36 | *r*=0.13  *p=*0.49 | **-2.1 (-3.9, -0.28)**  ***p*=0.02** | **-2.3 (-4.5, -0.15)**  ***p*=0.03** | 0.36 (-1.2, 1.9)  *p*=0.64 | 0.67 (-0.93, 2.3)  *p*=0.40 |
| NS | *r*=0.32  *p=*0.08 | *r*=0.28  *p=*0.14 | **-3.2 (-6.0, -0.55)**  ***p*=0.02** | **-3.7 (-6.6, -0.73)**  ***p*=0.01** | 0.28 (-2.2, 2.8)  *p*=0.82 | 1.2 (-1.2, 1.9)  *p*=0.64 |
| 0S | *r*=0.32  *p=*0.08 | *r*=0.26  *p=*0.16 | -3.1 (-7.2, 1.1)  *p*=0.14 | -3.3 (-7.2, 0.54)  *p*=0.09 | 0.61 (-2.6, 3.9)  *p*=0.70 | 1.8 (-1.4, 5.0)  *p*=0.27 |
| 2S | *r*=-0.04  *p=*0.84 | *r*=-0.09  *p=*0.65 | -1.4 (-4.75, 1.8)  *p*=0.37 | -1.5 (-4.75, 1.7)  *p*=0.34 | -0.63 (-3.1, 1.8)  *p*=0.60 | 0.17 (-2.3, 2.7)  *p*=0.89 |
| 6S | *r*=0.30  *p=*0.11 | *r*=0.26  *p=*0.16 | **-2.6 (-5.0, -0.22)**  ***p*=0.03** | **-2.9 (-5.7, -0.20)**  ***p*=0.03** | 0.25 (-1.7, 2.2)  *p*=0.80 | 0.77 (-1.2, 2.8)  *p*=0.44 |
| *N*-Sulfated^e^ | *r*=0.22  *p=*0.25 | *r*=0.18  *p=*0.35 | **-2.6 (-4.8, -0.36)**  ***p*=0.02** | **-2.8 (-5.3, -0.18)**  ***p*=0.03** | 0.22 (-1.7, 2.1)  *p*=0.71 | 0.81 (-1.1, 2.7)  *p*=0.40 |
| **Proteoglycan^a^** |  |  |  |  |  |  |
| Syndecan-1 | ***r*=0.57**  ***p<*0.01** | ***r*=0.52**  ***p*<0.01** | 0.33 (-1.7, 2.4)  *p*=0.74 | -0.64 (-3.1, 1.8)  *p*=0.59 | -0.69 (-2.4, 1.1)  *p*=0.43 | 0.26 (-1.2, 1.7)  *p*=0.70 |

EGCX = endothelial glycocalyx; GAG = glycosaminoglycan; HS = heparan sulfate; PARDS = pediatric acute respiratory distress syndrome; PELOD-2 = Pediatric Logistic Organ Dysfunction Score 2; PICU LOS = pediatric intensive care unit length of stay; VFD = ventilator-free days.

^a^ Biomarker data was log_10_ transformed.

^b^ Correlations were assessed with Pearson’s correlation coefficient (*r*).

^c^ Linear regression assessed the relationship between biomarker data (per log_10_ increase) and VFDs at 28 days and PICU LOS with regression coefficient [β] and 95% confidence interval (CI) displayed.

^d^ Models were adjusted for age, sex, immunocompromised status, and PRISM-III score

^e^ *N-*sulfated HS subtypes include NS2S, NS6S, and NS HS disaccharides

Bold = statistical significance *p*<0.05

**(A) (B)**

*r*=0.21

*p*=0.26

*r*=0.63

**p*<0.01

**Supplemental Figure 3.** Correlation of syndecan-1 with (**A**) heparan sulfate and (**B**) chondroitin sulfate among sepsis-associated pediatric acute respiratory distress syndrome (PARDS) patients. Biomarker data were log_10_ transformed and assessed with Pearson’s correlation coefficient (*r*).
